# Diet Rich in Animal Protein Promotes Pro-inflammatory Macrophage Response and Exacerbates Colitis in Mice

**DOI:** 10.3389/fimmu.2019.00919

**Published:** 2019-04-26

**Authors:** Klara Kostovcikova, Stepan Coufal, Natalie Galanova, Alena Fajstova, Tomas Hudcovic, Martin Kostovcik, Petra Prochazkova, Zuzana Jiraskova Zakostelska, Martina Cermakova, Blanka Sediva, Marek Kuzma, Helena Tlaskalova-Hogenova, Miloslav Kverka

**Affiliations:** ^1^Laboratory of Cellular and Molecular Immunology, Institute of Microbiology of the CAS, v.v.i., Prague, Czechia; ^2^Laboratory of Cell and Developmental Biology, Institute of Molecular Genetics of the CAS, v.v.i., Prague, Czechia; ^3^Laboratory of Gnotobiology, Institute of Microbiology of the CAS, v.v.i., Nový Hrádek, Czechia; ^4^Laboratory of Fungal Genetics and Metabolism, Institute of Microbiology of the CAS, v.v.i., Prague, Czechia; ^5^Laboratory of Molecular Structure Characterization, Institute of Microbiology of the CAS, v.v.i., Prague, Czechia; ^6^Faculty of Applied Sciences, University of West Bohemia, Pilsen, Czechia; ^7^Department of Pharmacology, Institute of Experimental Medicine of the CAS, v.v.i., Prague, Czechia

**Keywords:** dietary protein, microbiota, colitis, germ-free, macrophage

## Abstract

Diet is a major factor determining gut microbiota composition and perturbances in this complex ecosystem are associated with the inflammatory bowel disease (IBD). Here, we used gnotobiotic approach to analyze, how interaction between diet rich in proteins and gut microbiota influences the sensitivity to intestinal inflammation in murine model of ulcerative colitis. We found that diet rich in animal protein (aHPD) exacerbates acute dextran sulfate sodium (DSS)-induced colitis while diet rich in plant protein (pHPD) does not. The deleterious effect of aHPD was also apparent in chronic DSS colitis and was associated with distinct changes in gut bacteria and fungi. Therefore, we induced acute DSS-colitis in germ-free mice and transferred gut microbiota from aCD or aHPD fed mice to find that this effect requires presence of microbes and aHPD at the same time. The aHPD did not change the number of regulatory T cells or Th17 cells and still worsened the colitis in immuno-deficient RAG2 knock-out mice suggesting that this effect was not dependent on adaptive immunity. The pro-inflammatory effect of aHPD was, however, abrogated when splenic macrophages were depleted with clodronate liposomes. This treatment prevented aHPD induced increase in colonic Ly-6C^high^ pro-inflammatory monocytes, but the ratio of resident Ly-6C^−/low^ macrophages was not changed. These data show that the interactions between dietary protein of animal origin and gut microbiota increase sensitivity to intestinal inflammation by promoting pro-inflammatory response of monocytes.

## Introduction

Commensal gut microbiota significantly influences the host's metabolism. Its catabolic and anabolic pathways allow it to use a broad spectrum of substrates including ingested items, substances secreted into the gut lumen or those directly produced by (other) microbes (1). Out of these sources, diet represents the major bulk, and since it is the easiest one to influence, dietary intervention of microbiota and health brings most attention. Changes in diet drive microbes to adapt to a new substrate, thus induce profound changes in microbiota that could improve the host's ability to adapt to the environment. The changes induced by dietary extremes may persist for a long time and thus reflect the host's diet (2). These adaptive changes are similar across different mammalian lineages and have important implications for host's health (3). Dietary interventions can also induce rapid and reproducible changes in microbiota and enrich the community with transient food-borne microbes that are not capable of long-time colonization (4). Indeed, ~15% of microbial operational taxonomic units (OTUs) show strong diurnal fluctuation due to the timing of food intake thus synchronize circadian clock down to the level of metabolic processes. Interestingly, long-term violation of this fine-tuned system in shift workers and frequent flyers could cause temporal dysbiosis with serious metabolic consequences (5).

Disruption of gut microbiota, i.e., dysbiosis, has been recently linked to pathogenesis of inflammatory bowel disease (IBD) (6), metabolic syndrome (7, 8), cardiovascular diseases (9), neurological disorders (10), and even cancer (11). There is an increasing body of evidence that dietary macronutrients may both promote or counteract dysbiosis and its consequences (12). Both short- and long-term dietary interventions lead to substantial changes in gut microbiota and can influence pro-inflammatory status of gut mucosa (13, 14). There is a well-recognized link between diet and IBD. Retrospective studies found that high intake of sucrose, red meat, or margarine increases the relative risk for IBD, consumption of cereal, fruit and vegetable or high-fiber food in general decreases it (15–18).

Increased dietary intake of animal proteins has been proposed as a factor contributing to the Crohn's disease development already several decades ago (19, 20). Metabolism of proteins mainly takes place in the distal colon where carbohydrate-based sources are reduced and bacteria can switch their metabolism to asaccharolytic one. Depending on the source of the protein, animal, and diary proteins are almost completely degraded during passage whereas plant proteins are less degraded and reach large intestine in bigger amounts (21). Degradation of proteins, peptides and amino acids leads to production of various biologically active metabolites, such as branched-chain fatty acids, ammonium, hydrogen sulfide, p-cresol, phenolic and indolic derivates, which could influence the epithelial cells viability and proliferation, thus affecting gut barrier function and immune response. Some of these activities have been linked to the pathogenesis of IBD and other gastrointestinal diseases (22).

Several studies analyzed the effect of diet rich in protein (HPD) on gastrointestinal physiology and basic epithelial response. Rats consuming HPD had different microbiota composition and metabolic activity, altered colonocytes morphology and enzymatic pathways, more goblet cells and increased production of mucin when compared to rats consuming normoproteic diet (23–25). Induction of experimental inflammation in mice fed by HPD led to severe colitis with high mortality (26). Recently, study analyzing the macronutrient source and quantity found that high amount of dietary casein most significantly contributes to colitis sensitivity while psyllium fiber sensitivity decreases. In both these cases, microbiota composition, and density and gut barrier functions were the major mechanisms involved (27). Taken together these studies showed that various dietary proteins can change host's response to gut microbiota including fine tuning of mucosal immune system and eventually influence the susceptibility to experimental colitis.

Products of intestinal microbiota metabolism (such as short-chain fatty acids, SCFA) influence T cell maturation and activity and may regulate the host's immune system both directly and indirectly via other cells (28, 29). By attaching to the gut epithelium, microbes can regulate the balance in gut T cell response. Depending on the particular microbe, they may induce either regulatory T cells or pro-inflammatory Th17 cells (30, 31). In either case, mononuclear cells in the gut mucosa play crucial role in mucosal immune response and in sensitivity to intestinal inflammation. In fact, mononuclear cells such as macrophages residing in the gut mucosa contribute to the local tolerance and non-inflammatory environment, by producing IL-10 and PGE_2_ and not responding to bacterial lipopolysaccharide (32). Whereas, Ly6C^+^ blood monocytes are recruited to the mucosa in response to pro-inflammatory stimulation; outnumbering resident macrophages and producing large quantities of IL-1β and TNF-α (33, 34).

The aim of this study was to analyze how dietary protein can contribute to the IBD pathogenesis, with special focus on microbial and immunological mechanisms using two sets (animal and plant protein-based) of fully synthetic normo- and hyper-proteic diets. The gnotobiotic experiments were employed to decipher the importance of host-diet-microbiota interaction and its consequences for acute intestinal inflammation.

## Materials and Methods

### Animals

Immuno-competent BALB/c and immuno-deficient RAG2 knock-out mice on BALB/c background were obtained from breeding colony of the Institute of Physiology of the CAS and Institute of Microbiology of the CAS, respectively, and all experiments were performed under either conventional or germ-free conditions at the Institute of Microbiology of the CAS. Mice were fed with Maintenance diet for rats and mice No. 1324 (Altromin Spezialfutter, GmbH & Co. KG, Germany), unless stated otherwise. Different experimental groups were housed in separate cages. In addition to that, germ-free mice were housed in Trexler-type plastic isolators under sterile conditions, supplied with sterile water for several generations before the experiments started. This study was carried out in accordance with the recommendations of the ethics standards defined by the EU legislation on the use of experimental animals (2010/63/EU) and Czech animal welfare act. The protocol was approved by the Institute of Microbiology animal care and use committee (approval ID: 85/2015 and 108/2016).

### Diets and Experimental Design

In most experiments, shortly after weaning, the mice were switched either to animal protein-based diet—control (aCD, 176 g/kg crude protein; Cat# C1000) and high-protein diet (aHPD, 514 g/kg crude protein; Cat# C1001) or plant protein-based diet—control (pCD, 173 g/kg crude protein; Cat# C 1000/110007) and high-protein diet (pHPD, 500 g/kg crude protein; Cat# C1001/1001127; all from Altromin Spezialfutter, GmbH & Co. KG). All these diets were prepared synthetically, containing either casein (animal) or wheat gluten (plant) as a protein source (Table 1). Mice were maintained on these diets for 3 weeks before the experiment started and then throughout the whole experiment. In some experiments, the long-time effect of the diet was analyzed by switching the diet already to the parental generation of the mice. We measured gut permeability for macromolecules in healthy mice fed with different diets. For this purpose, we treated mice orally with 440 mg/kg of body weight of FITC-labeled 3–5 kD molecule of dextran (Merck, Cat# FD4), and measured serum fluorescence 4 h later, as we published previously (35).

**Table 1:** Composition of the experimental diets.

	**aCD**	**aHPD**	**pCD**	**pHPD**
Protein [g/kg]	176.115	514.115	172.685	500.440
Fat [g/kg]	50.830	51.030	70.937	70.002
Fiber [g/kg]	40.450	39.370	30.358	30.490
Ash [g/kg]	54.943	63.343	53.100	51.522
Moisture [g/kg]	81.736	77.736	76.874	71.076
Mono and disaccharides [g/kg]	110.960	110.960	110.650	56.806
Polysaccharides [g/kg]	471.700	115.700	422.425	116.147
Energy [kcal/kg]	3518.055	3458.055	3641.566	3669.700

### Acute and Chronic Experimental Colitis

Acute and chronic experimental colitis was induced by 3% dextran sulfate sodium (DSS, 36–50 kDa; MP Biomedicals, CA, USA; Cat# 02160110) solution in sterile tap water *ad libitum* (36). Acute colitis was evaluated on the last day of the experiment (day 8) by using a disease activity index (DAI), colon length and histological scoring system, as described previously (37). DAI was determined as a mean of the following three parameters: weight loss (none 0, 5% 1 point, 5–10% 2 points, 10–15% 3 points, >15% 4 points) stool consistency (solid 0 points, loose stool that do not stick to the anus 2 points, and 4 points for liquid stools that stick to the anus), rectal bleeding (none 0, positive test for occult blood 2 points, and 4 points for gross bleeding). Occult blood in feces was evaluated with Fecal Occult Blood Test (Okult-viditest Rapid; Vidia, Vestec, Czech Republic), which is based on guaiacum reaction. Postmortem, the mesenteric lymph nodes, spleen, and colon were collected from each mouse for further analyses. Colon shortening is an indirect marker of colitis severity, so the entire colon was removed (from caecum to anus) and measured by placing it without tension on a ruler. Next, colon descendens was collected for histological analysis, as described previously. Briefly, tissues were fixed in 4% formalin, dehydrated in ethanol and embedded in paraffin. Four micrometer sections were rehydrated and stained by hematoxylin and eosin. Subsequent microscopic evaluation was made by experienced pathologist in blinded manner.

Chronic DSS colitis was induced by three cycles consisting of 5 days DSS and 9 days tap water. The analyses of colitis severity by DAI, colon shortening, and mucosal damage were performed as described above. The level of acute-phase protein haptoglobin was determined in mouse serum using the mouse haptoglobin ELISA Duoset (Bio-Techne, Minneapolis, MN, USA; Cat# DY4409).

### Macrophages Depletion *in vivo*

Two days before DSS treatment, the mice were intraperitoneally injected with 200 μl of empty liposomes or liposomes loaded with clodronate (Liposoma BV, Amsterdam, The Netherlands; Cat# CP-010-010) to deplete blood monocytes and possibly some of tissue macrophages in spleen and colon (38). To maintain and promote the depletion through the whole DSS treatment period, we injected the mice every 3 days starting 2 days before the DSS introduction (day −2, day 1, and day 4).

### Tissue Cell Cultures and Cytokines Measurement

Colon tissue was cultivated *ex vivo* as described earlier (37). Briefly, three millimeter punch biopsy from distal colons were collected, weighted and cultured in 500 μl of complete RPMI medium (Merck; Cat# R0883) containing 10% heat-inactivated fetal bovine serum (Biochrom GmbH, Germany; Cat# S 0115) and 1% Antibiotic-Antimycotic solution (Merck) in humidified incubator (37°C, 5% CO_2_) for 48 h. The supernatants were collected and stored at −20°C until analysis. Cytokines were measured in tissue culture supernatants using appropriate ELISA sets (Bio-Techne; Cat# DY410, DY406, DY401, and DY3626) according to the manufacturer's instructions.

### Cell Preparation and Flow Cytometry Analysis

Single cell suspensions from mesenteric lymph nodes (mLN) and spleens were prepared by mechanical disruption and passed through a 70 μm cell strainer (Becton Dickinson; Cat# 352350). After the washing (300 × g, 5 min, 4°C), red blood cells from spleens were lysed by 5 min incubation in RBC lysing buffer (1 mM EDTA, 150 mM NH_4_Cl, 10 mM KHCO_3_). The supernatant with lysed red blood cells was removed and cells were used for further analyses. Single cell suspensions from colons were prepared using the published protocol (39). Next, the cells were blocked by normal mouse serum and incubated with fluorochrome-conjugated antibodies recognizing extracellular epitopes (Supplementary Table 1). Then, the cells were treated with eBioscience™ Foxp3/Transcription Factor Staining Buffer Set (Thermo Fisher Scientific; Cat# 00-5523-00) and stained for intracellular antigens (Supplementary Table 1). To distinguish viable and dead cells, Fixable Viability Dye—eFluor 780 (Thermo Fisher Scientific; Cat# 65-0865-18) was added to the staining mix before fixation. Data were obtained by measuring the samples on LSRII (BD Biosciences, CA) and the FlowJo software (Tree Star Inc., Ashland, OR; RRID: SCR_008520) was used for data analyses. Example of used gating strategy is shown on Supplementary Figure 7.

### Real-Time PCR

Three millimeter punch biopsy from distal colons were collected, weighted, immediately immersed in RNA*later* RNA Stabilization Reagent (Qiagen, Hilden, Germany; Cat# 76106) and stored at −20°C until RNA extraction. Next, the tissue was homogenized with ceramic beads (Lysing matrix D; MP Biomedicals, Santa Ana, CA, USA; Cat# 116913050) using FastPrep-24 (MP Biomedicals) and total RNA was isolated using TRI reagent (Zymo Reseaerch, Irvine, CA, USA; Cat# R2050). Next, the samples were treated with DNAse (TURBO DNA-free™ Kit; ThermoFisher Scientific; Cat# AM1907) according the manufacturer's instructions, and then reverse transcribed into cDNA with the SuperScript IV Reverse Transcriptase and the RNaseOUT™ Recombinant Ribonuclease Inhibitor (ThermoFisher Scientific; Cat# 10777019), in the presence of random primers (Generi Biotech s.r.o., Hradec Králové, Czech Republic). The resulting cDNA was used for quantitative PCR (CFX96 Touch™, Bio-Rad) with gb SG PCR Master Mix (Generi Biotech s.r.o.; Cat# 3005). Before use, each set of primers (Supplementary Table 2) and RT-PCR conditions were extensively optimized to ensure high efficiency of the reaction and to avoid primer dimers. The cycling parameters were as follows: 3 min at 95 °C, 40 cycles of 30 s at 94 °C, 40 s at 59 °C, and 50 s at 72 °C. Quantitative RT-PCR data were invariably normalized to the expression levels of the reference gene ribosomal protein S12 (Rps12) by means of the 2^−ΔΔCt^ method.

### Gut Microbiota Analysis

Stool samples were collected at three different time points: before switching to experimental diets (day −21), before disease induction (day 0) and at the termination of the experiment (day 8). Total DNA was extracted using MasterPure Complete DNA and RNA Purification Kit (Epicenter, Illumina Inc., Madison, WI, USA; Cat# MC85200) with repeated bead-beating in Lysing Matrix Y tubes using FastPrep homogenizer (both MP Biomedicals) and PCR inhibitors were removed using InhibitEx Tablets (Qiagen; Cat# 19590). DNA was then standardized using Qubit dsDNA High Sensitivity kit (Thermo Fisher Scientific). PCR targeting V3 and V4 regions of bacterial 16S was conducted using Kapa HiFi DNA polymerase (Kapa Biosystems, Wilmington, MA, USA), primers 341F (5′-CCTACGGGNGGCWGCAG- 3′) and 806R (5′-GGACTACHVGGGTWTCTAAT- 3′) (40) (Generi Biotech s.r.o.) and 10% BSA (Merck; Cat# 8894). Cycling conditions consisted of initial denaturation (94°C, 3 min) followed by 30 cycles of denaturation (94°C, 30 s), annealing (54.2°C, 45 s), extension (72°C, 75 s) and final extension (72°C, 10 min). For PCR targeting fungal ITS1 region, PPP Master Mix (Top-Bio, Vestec, Czech Republic; Cat# P126) with 18SF (5′-GTAAAAGTCGTAACAAGGTTTC-3′) and 5.8SR (5′-GTTCAAAGAYTCGATGATTCAC-3′) primers and 10% BSA was used (41). Cycling conditions were 95°C, 5 min; 35 cycles of 95°C, 30 s; 50°C, 30 s; 72°C, 60 s, and 72°C, 10 min. PCR triplicates were pooled and purified by SequalPrep Normalization Plate Kit (Thermo Fisher Scientific; Cat# A1051001). Samples within library were pooled and sequencing adaptors were ligated using TruSeq DNA PCR-free LT Sample Preparation Kit (Illumina, Madison, WI, USA; Cat# FC-121-3001). Ligated libraries were quantified with KAPA Library Quantification Kit (Kapa Biosystems, Wilmington, MA, USA; Cat# KK4824) and sequenced on MiSeq Illumina Platform using Miseq Reagent Kit v3 (Illumina; Cat# MS-102-3003) at The Genomics Core Facility, CEITEC (Brno, Czech Republic). Sequencing data were processed using QIIME version 1.9.1 (42). Quality filtering, chimera detection and read demultiplexing and read clustering were done as described previously (43). Fungal reads were in addition extracted for ITS1 region using ITSx package (44). Identification of representative sequences was done using RPD classifier (45) against bacterial GREENGENES database 13.8 (46) and fungal UNITE database 7.2 (UNITE Community (2017): UNITE QIIME release. Version 01.12.2017. UNITE Community. https://doi.org/10.15156/BIO/587481). Finally OTU table was produced. The data are available in the Sequence Read Archive (SRA) http://www.ncbi.nlm.nih.gov/sra under the submission number SUB4976849. Briefly, for microbiota analysis, Chao1 index was used to describe alpha diversity and Principle Coordinate Analysis (PCoA) based on Bray Curtis dissimilarity metrics was used to describe beta diversity. Next, Linear discriminant analysis effect size (LEfSe; RRID: SCR_014609) was used to determine the typical taxonomic profiles of communities in each group (42, 47). Functional composition of a bacterial metagenome was predicted by PICRUSt tool, using the 16S amplicon data (48).

### Gut Microbiota Metabolomics

At day 0, one fecal pellet of ~25 mg from each mouse was collected and homogenized by vigorous vortexing in 500 μl of phosphate buffered saline (PBS; pH = 7.4). Next, the sample was centrifuged (13,000 × g for 10 min) to remove particulate matter and supernatant was transferred to a fresh microfuge tube and centrifuged again. The resulting supernatant was lyophilised at −58°C overnight, re-suspended in D_2_O (500 μl) containing 0.01% trimethylsilyl propionic acid as an internal standard and transferred to a 5-mm NMR tube. The NMR spectra were recorded on a 600 MHz Bruker Avance III spectrometer (Bruker BioSpin, Rheinstetten, Germany) equipped with a 5-mm TCI cryogenic probe head. More detailed description of experimental conditions of NMR analysis and corresponding processing steps is given in Supplementary information. Total protein content in fecal pellet filtrates was measured by the bicinchoninic acid (BCA) assay (Pierce™ BCA Protein Assay Kit; ThermoFisher Scientific; Cat# 23227) according to manufacturer's recommendations.

### Statistical Analysis

One-way analysis of variance (ANOVA) with Tukey's multiple comparison test was used to compare multiple experimental groups. Two-way ANOVA with Bonferoni *post-test* was used in determination of significant weight and DAI changes. Differences between two groups were evaluated using an unpaired two-tailed Student's *t*-test. The data are presented as the mean ± standard deviation and differences were considered statistically significant at *P* ≤ 0.05. GraphPad Prism statistical software (version 5.0, GraphPad Software, RRID: SCR_002798) was used for analyses.

## Results

### Protein-Rich Diet of Animal Origin Worsen Acute Colitis While Protein-Rich Diet of Plant Origin Does Not

**Figure 1:**
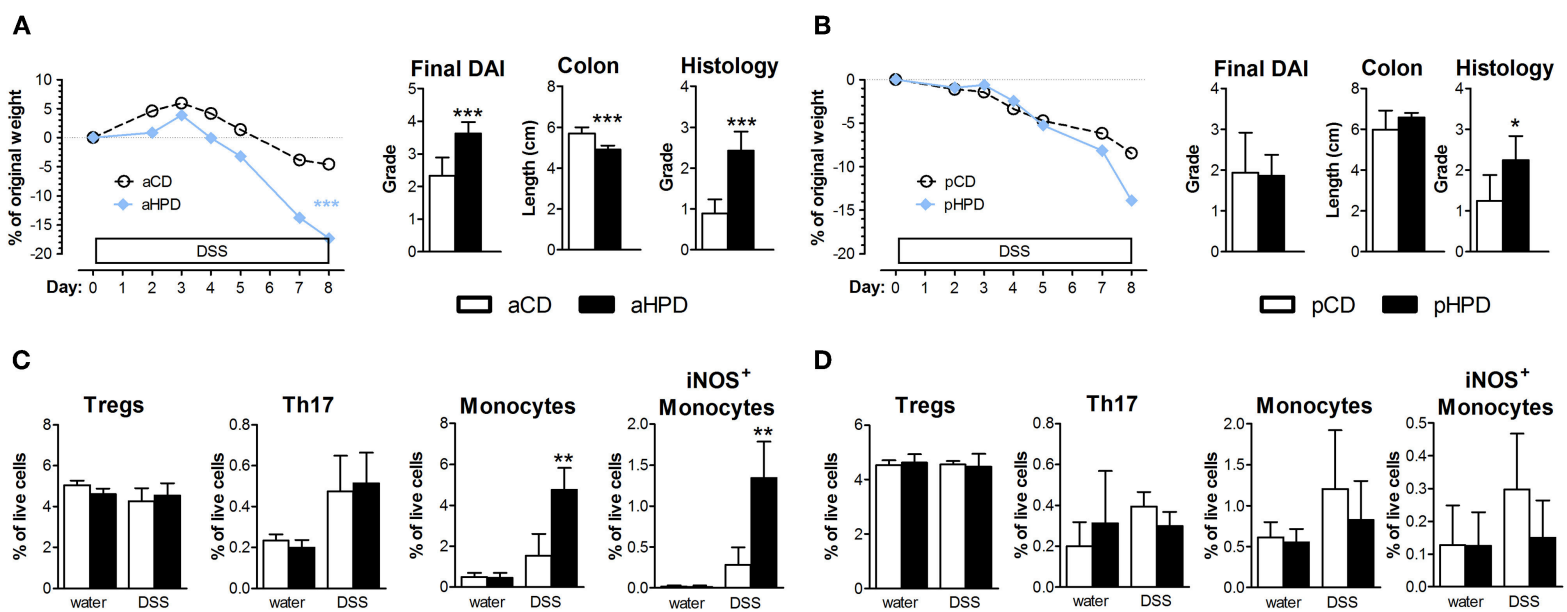
In immune-competent BALB/c mice, HPD of animal origin significantly worsens the severity of acute DSS-colitis **(A)** while HPD of plant origin does not **(B)**. Colitis severity was measured by disease activity index (weight loss, stool consistency, and rectal bleeding), colon shortening and damage to colon mucosa. Neither aHPD **(C)** nor pHPD **(D)** changed the proportions of regulatory T cells (CD3^+^CD4^+^FoxP3^+^) or Th17 cells (CD3^+^CD4^+^RorγT^+^) in mesenteric lymph nodes, while monocytes (CD11c^low^Ly-6C^hi^) were significantly increased and more activated (iNOS^+^) in colons of aHPD fed mice. The weight loss was analyzed by two-way ANOVA and other parameters by unpaired Student's *t*-test; ^*^p < 0.05 ^**^p < 0.01 ^***^p < 0.001. Data are from one representative experiment out of 6 **(A,C)** or 3 **(B,D)** independent experiments (*n* = 5–8).

To analyze the effect of quantity and source of dietary protein on acute intestinal inflammation, we exposed mice fed with animal or plant protein-based diets to DSS. We found that the resulting colitis was significantly more severe in animals fed with diet rich in animal protein (aHPD) than in animals fed with diet containing normal amount of animal protein (aCD) (Figure 1A). On the other hand, mice fed with diet rich in plant protein (pHPD) were only marginally more susceptible to acute colitis than animals fed with diet containing normal amount of plant protein (pCD) (Figure 1B). Neither animal protein- (Figure 1C) nor plant protein-based diets (Figure 1D) influenced the proportions of regulatory T cells or Th17 cells in mesenteric lymph nodes. But unlike the increase in plant protein, increase in animal protein led to significant increase in colonic Ly-6C^hi^ monocytes and their activation. The intestinal inflammation in aHPD mice was accompanied with increased colonic expression of anti-inflammatory TGF-β, pro-inflammatory cytokines (TNF-α and IL-1β) and inducible NO synthase. In the similar situation, pHPD generally decreased their expression. Neither aHPD nor pHPD influenced the colonic expression of IL-18 (Supplementary Figure 1). Furthermore, we found that neither aHPD nor pHPD significantly changed animal growth, gut permeability for macromolecules or gut cytokine production in healthy mice (just before the DSS exposure) (Supplementary Figure 2).

Since short term dietary change may have different consequences than the whole life dietary change, we changed diet to mice before mating and induced acute DSS colitis in their offsprings. This way, we ensured that the mice were kept on the aCD or aHPD their entire life. Nevertheless, even in this setup, high amounts of animal protein significantly increased sensitivity to acute DSS colitis (Supplementary Figure 3).

### The aHPD Worsens the Intestinal Inflammation in Chronic Colitis

**Figure 2:**
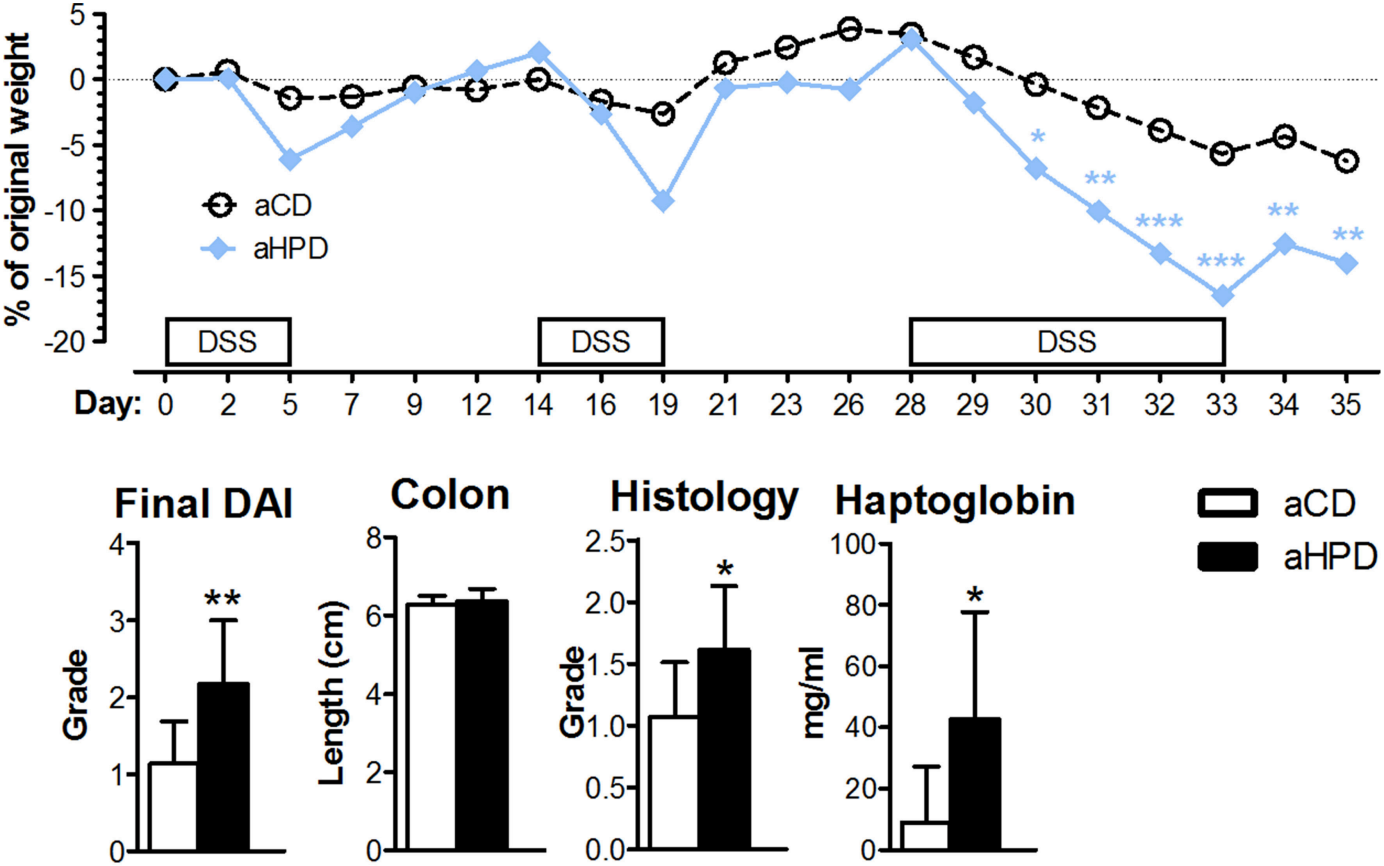
HPD of animal origin worsens the severity of chronic DSS-colitis. The weight loss was analyzed by two-way ANOVA and other parameters by unpaired Student's *t*-test; ^*^p < 0.05 ^**^p < 0.01 ^***^p < 0.001. Data are from one representative experiment out of 2 independent experiments (*n* = 5–8).

Next, we induced chronic colitis in aCD and aHPD mice by repeated administration of DSS. We found that aHPD worsened the course of chronic DSS colitis. As shown on the weight curve, every cycle of DSS treatment led to a more pronounced inflammation in the aHPD-fed group (Figure 2).

### Gut Microbiota Is Significantly Altered in Both aHPD and pHPD-Fed Mice

**Figure 3:**
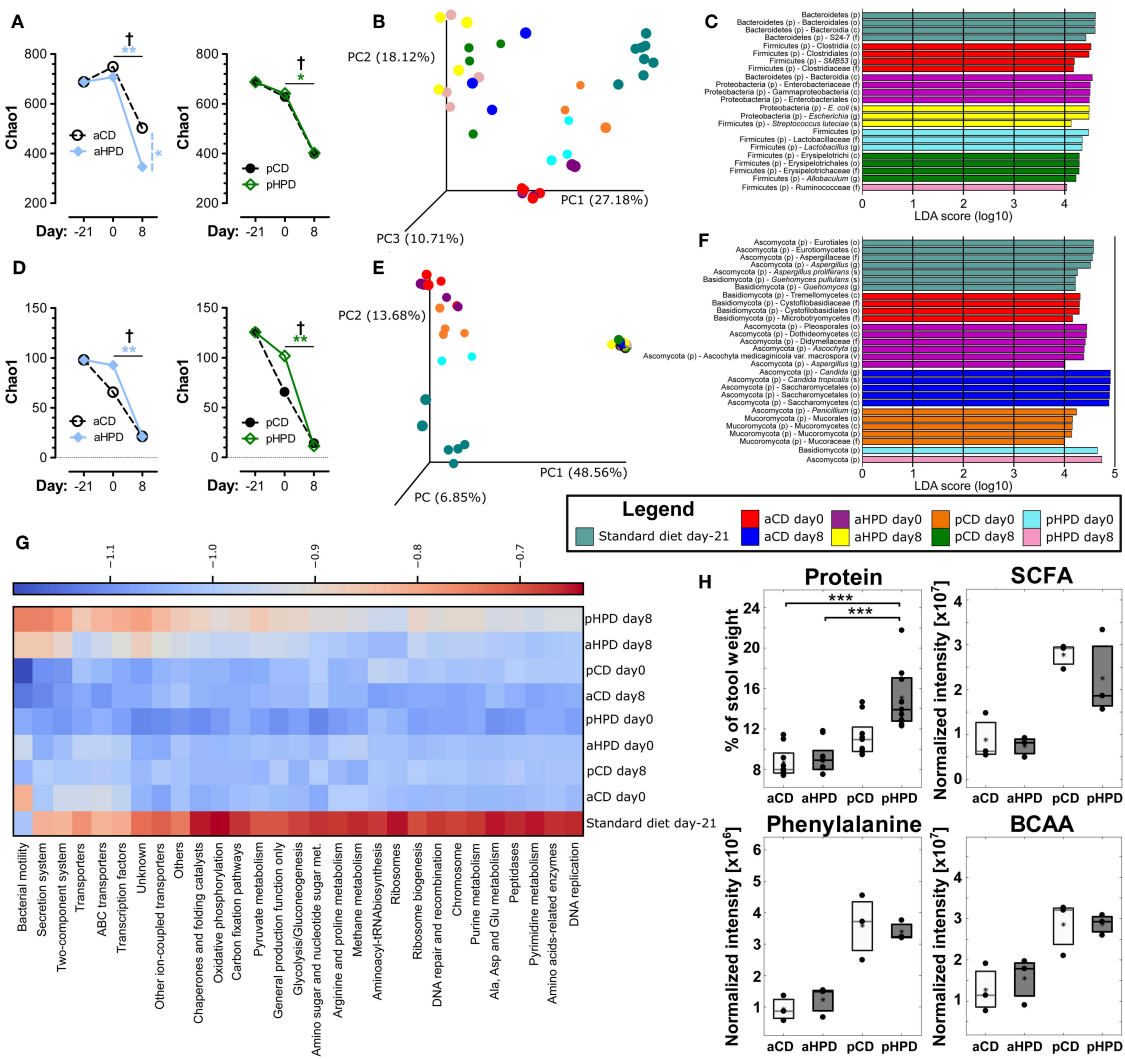
Gut microbiota, both bacterial and fungal, is profoundly changed by diet and intestinal inflammation. Comparison of impact of diet and gut inflammation on bacterial **(A)** and fungal **(D)** alpha diversity using Chao1 diversity index. Principal coordinates analysis (PCoA) plot using the Bray-Curtis distance metric shows the compositional differences induced by diet and inflammation at the diet switch (day −21) and before (day 0) and after the DSS treatment (day 8) for bacteria **(B)** and fungi **(E)**. Each colored orb represents the microbiota composition in feces of one mouse. Each color represents one group of mice at the day −21, 0, or 8. Linear discriminant analysis effect size (LEfSe) for bacteria **(C)** and fungi **(F)** shows taxonomic units typical for each group. The first taxonomic unit represents phylum (p) and the second represents the lowest taxonomic rank revealed by the analysis, it being class (c)—order (o)—family (f)—genus (g)—species (s) or variety (v). Heatmap of bacterial gene functional predictions using the PICRUSt algorithm showing the changes in KEGG level-3 pathways (columns) and experimental groups (row) **(G)**. Fecal protein content, as measured by BCA assay and amounts of SCFA, phenylalanine and BCAA, as measured by NMR-based metabolomics **(H)**. The changes in alpha diversity were analyzed by ANOVA with Tukey's multiple comparison test; †*p* < 0.05 for changes in aCD or pCD and ^*^p < 0.05 ^**^p < 0.01 for the other comparisons. The metabolomic data were analyzed by Wilcoxon-Mann-Whitney test; ^*^p < 0.05 ^**^p < 0.01 ^***^p < 0.001 (*n* = 3). Boxplot legend: asterisk—mean value, black dots—metabolites' intensities in particular samples.

Changing the diet from standard chow to synthetic diets, either plant or animal protein-based, did not significantly changed bacterial alpha diversity, but DSS-induced colitis led to significant decrease in bacterial alpha diversity regardless of the diet (Figure 3A). Interestingly, aHPD decreased bacterial diversity significantly more than aCD (*p* < 0.05), but there was no similar effect in plant-based diets. The PCoA plot showed clear clustering among different diets and treatments among bacteria (Figure 3B). First, there was a clear shift between day −21 and 0, induced by the diet change, which was more significant in both animal protein-based diets than in the plant protein-based ones. As expected, the protein source seemed to be more important for the bacterial community composition than the protein amount. This clustering became less clear at day 8, when DSS-induced colitis seemed to have the major impact on bacteria. There were particular bacterial taxa responsible for these shifts (Figure 3C). Animal protein-based diets led to increased relative abundance of genera *Enterococcus, Streptococcus, Turicibater* (only aCD) and *Escherichia*, and families Peptostreptococcaceae and Ruminococcaceaea. On the contrary, plant protein-based diets enriched OTUs belonging to families Bifidobacteriaceae and Desulfovibrionaceae in pCD and Coriobacteriaceae in pHPD and both showed increased abundance of lactobacilli and families Lachnospiraceae and Erysipelotrichaceae. DSS treatment resulted in significant shifts in genera *Bacteroides* and *Parabacteroides* no matter which protein source was used. In addition, we found significant increase of *Escherichia coli* also in all groups; most notably in both HPDs. It was enriched from 2.02, 1.01, 0.04, and 0.27 to 10.03, 38.03, 17.34, and 34.03% in aCD, aHPD, pCD, and pHPD, respectively. Compared to other groups, we found some specific changes caused by colitis, such as more OTUs assigned to genus *Odoribacter* and *Akkermansia muciniphila* in mice consuming aHPD, and genus *Staphylococcus* in both HPDs. In mice consuming plant protein-based diets, colitis increased the relative abundance of genus *Enterococcus* from 0.03 and 0.02% to 1.06 and 9.57% in pCD and pHPD, respectively.

Similarly as in bacteria, there was some decreasing tendency in fungal alpha diversity in both CDs with the most striking decrease in fungal diversity during colitis induction (day 0 vs. 8) (Figure 3D). The beta-diversity shifts induced by diet and the effect of DSS-induced colitis were even more prominent for fungi (Figure 3E) than for bacteria. These changes were driven by marked shift in Ascomycota/Basidiomycota ratio before and after DSS treatment (from 62.49, 51.56, 55.28, and 327.03 to 3513.00, 5087.29, 3693.16, and 27738.64 for aCD, aHPD, pCD, and pHPD, respectively). Mice consuming animal protein-based diets showed increased relative abundance of families Cystofilobasidiaceae, Sporidiobolaceae, and Microbotryomycetes fam. incertae sedis. On the other hand, plant protein-based diets led to increased abundance of order Pleosporales and genera *Piskurozyma, Cryptococcus, Ascochyta*, and *Aspergillus* (Figure 3F). After DSS treatment, *Candida tropicalis* seemed to outgrow all other fungal species in all groups (from 0, 0, 0.3, and 1.6% to 94.9, 94.3, 93.7, and 88.5% in aCD, aHPD, pCD, and pHPD, respectively).

These taxonomic changes were reflected by the marked shifts in microbial function, as predicted by PICRUSt algorithm (Figure 3G). Almost all detected metabolic pathways or functional proteins seemed to be enriched in microbiota at day −21 when the mice were fed standard diet when compared to synthetic diets. The dietary change led to a tendency to reduce some pathways especially in plant protein-based diets at day 0 and partly also in aCD at day 8. Among proteins with the highest reduction belonged “bacterial motility proteins,” “secretion system” and “two-component system.” Interestingly, these pathways were slightly enriched after DSS treatment on day 8 in aHPD and pHPD. Moreover, overall pathways enrichment was visible at both HPDs on day 8 partly getting a pattern similar as at day −21.

### Metabolomics Reveals Shifts Between Animal and Plant Protein Diets

We compared the fecal metabolite profile among healthy mice (day 0) on different diets to better understand the luminal environment that could affect the sensitivity to intestinal inflammation. We found that gluten was more resistant to digestion than casein, which led to an increase in stool protein content, especially in pHPD (Figure 3H). Next, we identified several distinct metabolites that may influence experimental colitis induction, using NMR-based approach. We found that while there were no major differences between matched normoproteic and hyperproteic diets, the animal and plant protein-based diets each led to production of different metabolites suggesting that the source, and not the amount, of protein was important. Total short-chain fatty acids (SCFA; acetate, propionate, butyrate) were increased in fecal content of mice fed with both gluten-based diets as compared to the casein-based ones. Nevertheless, there were no significant differences in SCFA related to protein amount. Similarly as SCFA, phenylalanine or total branched-chain amino acids (BCAA; valine, leucine, isoleucine) were also higher in plant protein-based diets and similar trends were also visible in glutamate, glutamine, citrate, threonine, and arabinose. Only cytidine showed any tendency for an increase in fecal content of animal protein fed mice (Supplementary Figure 4).

### Both Microbiota and aHPD Are Needed for the Pro-inflammatory Effect of aHPD

**Figure 4:**
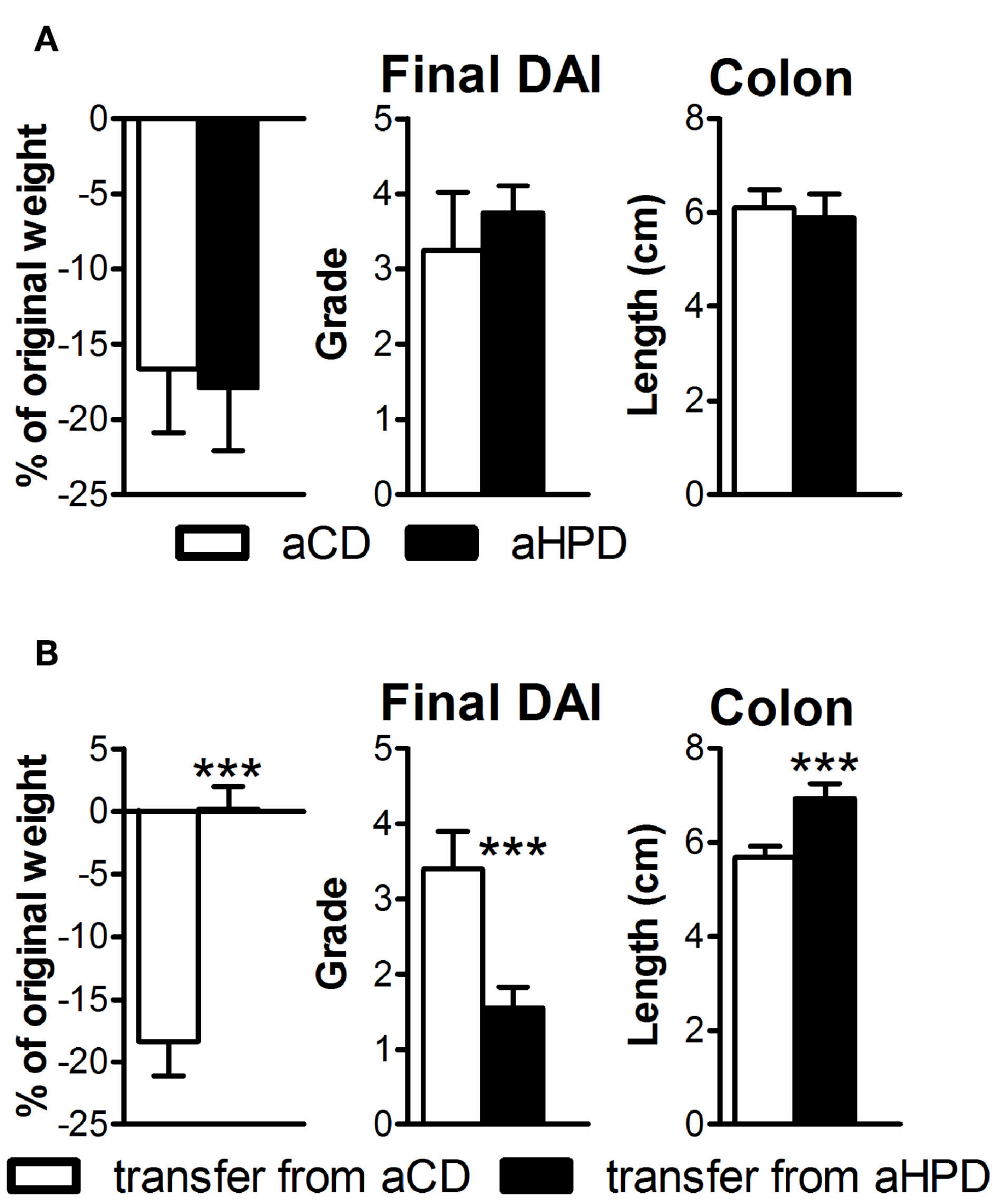
HPD of animal origin does not worsen the severity of acute DSS-colitis in germ-free animals **(A)**, but microbiota transferred from aHPD fed mice mitigates colitis severity **(B)**. The weight change shows percentage of weight gain/reduction between day 0 and 8 of DSS treatment. All parameters were analyzed by unpaired Student's *t*-test; ^***^p < 0.001 (*n* = 5–8).

First, we induced acute intestinal inflammation in germ-free (GF) mice on different diets, to find that both aCD and aHPD had similar severity of colitis (Figure 4A). Then we transferred gut microbiota from either healthy aCD-fed or healthy aHPD-fed mice to GF mice fed standard diet. Surprisingly, we found that microbiota transferred from aHPD-fed mice protected the mice from acute colitis (Figure 4B).

### The Pro-inflammatory Effect of aHPD Is Not Dependent on Adaptive Immunity

**Figure 5:**
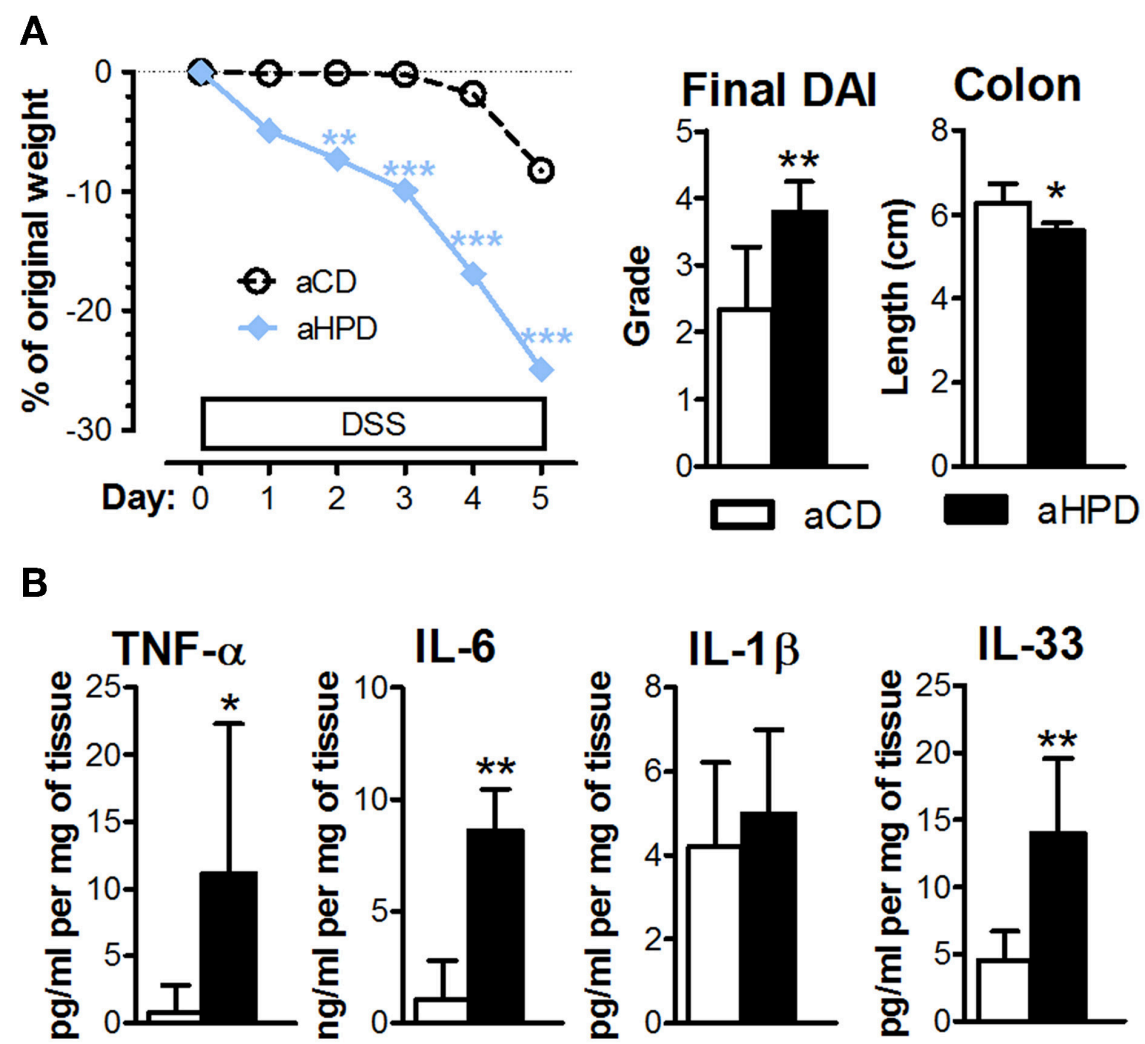
In immune-deficient RAG2^−/−^ mice, aHPD worsens the severity of acute DSS-colitis **(A)**, which is accompanied with the increase in TNF-α, IL-6, IL-1β, and IL-33 in the colon **(B)**. The weight loss was analyzed by two-way ANOVA and other parameters by unpaired Student's *t*-test; ^*^p < 0.05 ^**^p < 0.01 ^***^p < 0.001. Data are from one representative experiment out of 3 independent experiments (*n* = 6–7).

The lack of any differences between aCD or aHPD mice in regulatory T cells and Th17 cells (Figure 1C) suggested that adaptive immunity was not responsible for the pro-colitic effect of aHPD. To gauge the importance of adaptive immunity in the pro-inflammatory effect of aHPD, we repeated experiments with acute DSS colitis in mice lacking both T and B lymphocytes (RAG2^−/−^). Similarly as in immuno-competent BALB/c mice, aHPD-fed RAG2^−/−^ mice were significantly more sensitive to the acute intestinal inflammation than aCD-fed mice (Figure 5A). Moreover, colons of aHPD mice produced more pro-inflammatory cytokines TNF-α, IL-6, and IL-33 as compared to aCD mice (Figure 5B). Although there was slightly higher weight loss in pHPD mice, they were not more sensitive to acute colitis than pCD mice (Supplementary Figure 5).

### Inflammation in aHPD Fed Mice Is Driven by Monocytes and Subsequent Pro-inflammatory Tuning of Innate Immunity

**Figure 6:**
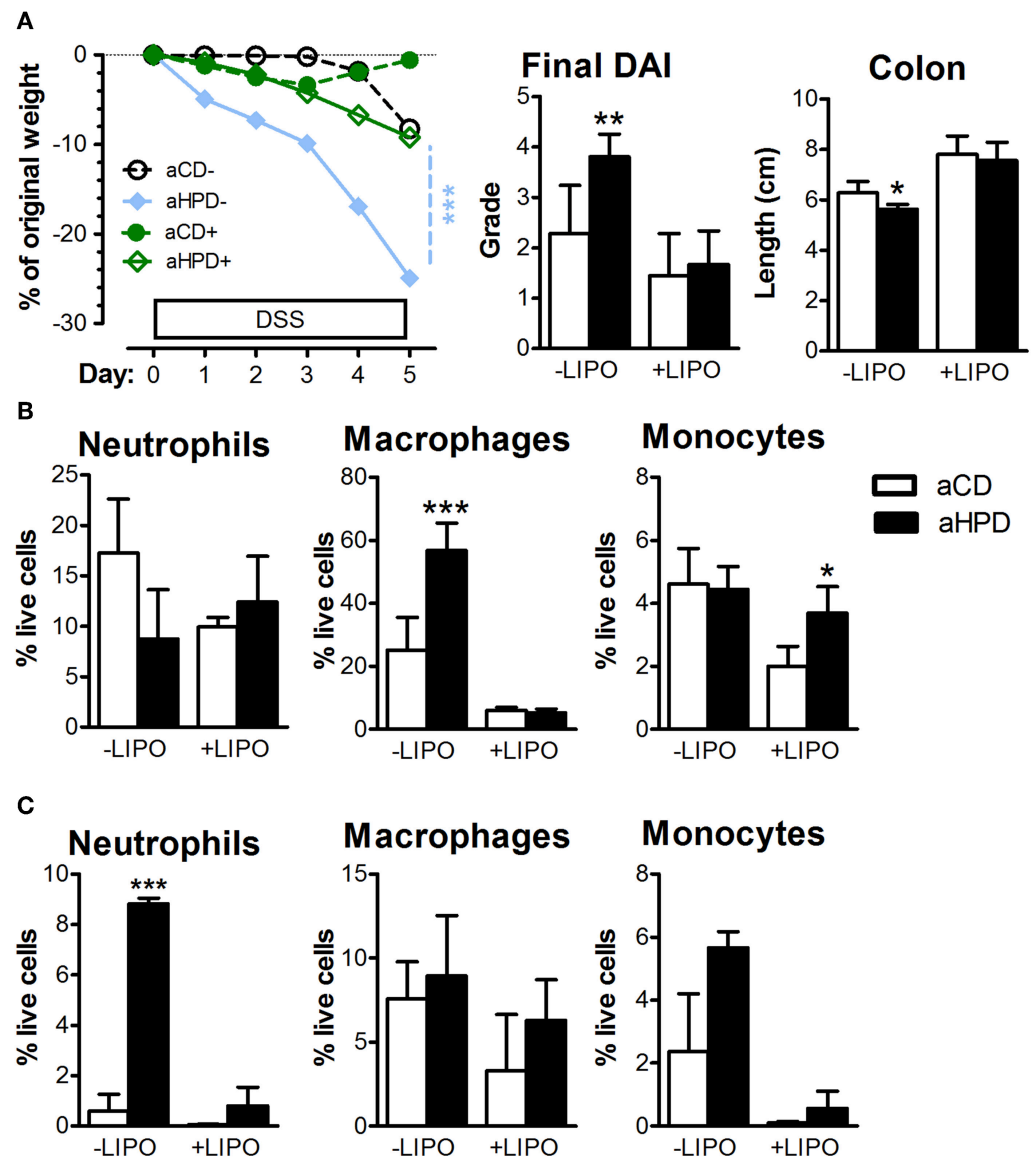
Intraperitoneal administration of clodronate liposomes is sufficient to mitigate the colitis in RAG2^−/−^ **(A)** mice. Splenic **(B)** and colonic **(C)** populations of neutrophils, macrophages and monocytes as analyzed by flow cytometry in colitic RAG2^−/−^ mice treated either with empty (–LIPO) or clodronate-loaded (+LIPO) liposomes. The weight loss was analyzed by two-way ANOVA and other parameters by unpaired Student's *t*-test; ^*^p < 0.05 ^**^p < 0.01 ^***^p < 0.001. Data are from one representative experiment out of 3 independent experiments (*n* = 6–7).

To analyze the importance of monocytes, we injected clodronate-containing liposomes (LIPO+) or empty liposomes (LIPO-) intraperitoneally every 3 days starting 2 days before the start of DSS. We found that this treatment abrogated the pro-colitic effects of aHPD in both BALB/c (Supplementary Figure 6) and RAG2^−/−^ (Figure 6A) mice. In RAG2^−/−^ mice, aHPD increased proportions of macrophages in spleen and neutrophils in colon (Figures 6B,C). LIPO+ treatment significantly reduced number of splenic Ly-6C^low^ macrophages and colonic Ly-6C^hi^ monocytes and neutrophils in aHPD-fed mice (*p* < 0.001 in all cases). Interestingly, while clodronate liposomes were capable to markedly reduce the proportions of macrophages in the spleen, they did not have such effect on colonic macrophages (Figure 6C).

## Discussion

Host's homeostasis is adjusted by continuous interactions between microbiota and immune system. Thus, change in microbiota caused by diet could lead to a shift in immune system reactivity and ultimately to changes in sensitivity to inflammatory diseases (49).

Here, we used animal model of ulcerative colitis to analyze how diet-microbiota-host interactions influence the sensitivity to intestinal inflammation. We found that HPD with protein of animal origin worsens acute colitis while HPD with protein of plant origin does not. Moreover, changing the diet 3 weeks before has similar outcome for the colitis as switching the diet already to the previous generation before conception. This suggests that increased sensitivity of mice fed aHPD is not a result of the slower adaptation of the organism to the fundamentally changed macronutrient ratio (13). While changes in dietary protein source in humans lead to profound changes in gut microbiota metabolism and host's gut mucosa transcription, casein-rich diet did not induce inflammation but influenced central homeostatic principles such as cell to cell signaling, cell cycle or death (50). This is in agreement with studies performed in IBD patients, where animal (especially milk) protein consumption correlated positively and consumption of plant protein negatively with incidence of Crohn's disease (20). In these cases, however, proteins in diets are often naturally accompanied with micronutrients with the ability to mitigate (51–53) or exacerbate (54–56) intestinal inflammation in animal models. To minimize the effect of these secondary bioactive molecules, we used well-matched and fully synthetic diets containing only one source of protein—casein or wheat gluten, respectively—in either normal (aCD/pCD) or high (aHPD/pHPD) amounts. The type and composition of other components was the same in all diets used. Diets of animal origin contain slightly more fiber (1% of total mass) and slightly less fat (2% of total mass). This, however, probably did not significantly influence the induction of colitis, because both fat and fiber content were similar between normo- and hyper-proteic diets of either type.

Our data support the recent study showing that high amounts of dietary protein accelerate carcinogenesis in AOM/DSS model of colon cancer by increase the colonic inflammation (57). Indeed, we confirmed that colons from colitic aHPD-fed mice produce more iNOS than colons of aCD-fed mice, but this difference seems to be more related specifically to casein than any protein. We found increased colonic expression of TGF-β, which forms one of the main regulatory feedback loop involving colonic macrophages and Tregs (33). Its increase during the more severe colitis in aHPD-fed mice may represent a local damage control and its suppressive effect on pro-inflammatory cells was probably counteracted by increased levels of pro-inflammatory cytokines, thus insufficient to regulate the inflammation in aHPD mice.

Main mechanism of acute DSS colitis is a damage to gut barrier function (58), but if it was compromised even before DSS exposure, it may accelerate colitis. We found that neither of the two high-protein diets significantly increased gut permeability for macromolecules in healthy mice, suggesting that the barrier was not compromised before the exposure to DSS. This is not in agreement with the study of Llewellyn et al. (27), who found that dietary casein increases gut permeability in healthy mice. We can speculate that this discrepancy was due to the better adaptation of our animals to the abrupt dietary change caused by the longer exposure (3 weeks instead of 1 week), different initial microbial colonization or differences in mouse strain (BALB/c instead of C57BL/6) used.

Diet is a major modulator of the gut intestinal microbiota thus can significantly influence the immune reactivity (14). There is a steep decrease in bacterial and fungal diversity as a result of DSS-induced colitis, which is typical feature shared with human UC (43). In the mice fed animal protein-based diets, we identified several bacterial genera, such as *Enterococcus, Streptococcus* and *Peptostreptococcus*, which have been formerly shown in association with IBD (59). Moreover, in colitic aHPD-fed mice, we found increased relative abundance of genera *Odoribacter* and *Akkermansia* which has been also linked to gastrointestinal diseases. Bacterial and fungal genera such as *Streptococcus, Peptostreptococcus, Escherichia* and *Candida* are known to express several virulence factors including proteolytic activity and biofilm formation that could improve their viability and adhesion in the gut and thus influence disease induction (60). While *Candida* mostly appeared after DSS course in all tested groups, streptococci, and peptostreptococci were present in the animal protein groups even before colitis induction and were massively outgrown with *E*. *coli* after DSS treatment. In our model, both *Candida* and *E. coli* were increased after feeding animal protein diet and thus they may facilitate detrimental effects of DSS treatment especially in aHPD group. These two microbes are often associated with diseased IBD patients (61, 62), and even their co-occurrence is necessary for severe forms of experimental colitis (63). Unfortunately, mechanisms of their cooperation are not yet known.

Prediction of proteins or metabolic pathways by PICRUSt analysis revealed some microbiota features that can be linked to increased susceptibility to gut inflammation. It has been shown that several strains of *E. coli* use flagella to improve their mobility in the gastrointestinal tract under acidic pH and in environment with higher levels of bile salts (64). Moreover, various transmembrane secretion systems are associated with pathobionts and pathogens in the gastrointestinal tract. Transport and secretion of metabolites including toxins can compromise the gut mucosa and even initiate inflammation (65). Two-component systems basically modulate microbial transcription in the way of quick adaptation to changing conditions thus having important role in bacterial survival. In our model, especially in HPDs, bacteria residing inflamed gut were equipped with these mechanisms improving their viability and promoting their possible pathogenicity.

By metabolomics analysis, we intended to define the conditions right before colitis induction and possibly connect them to the subsequent colitis course. Known for mostly beneficial effects, we determined the levels of SCFA. Though they are typically produced from saccharides, recent research has shown that also protein can serve as a substrate for their production (66). Nevertheless, when we compared animal and plant proteins intestinal digestion we found that animal protein derived the smallest levels of SCFA while plant protein showed quite high production. This could be associated with differences in endogenous digestibility of animal and plant proteins. In line with others (21, 22), our results are showing the later one is less digestible and thus available for further processing in the colon. Moreover, we found that plant protein diet led to milder colitis though some effect of DSS treatment was still visible. Since butyrate, as a main component of SCFA, has been shown to reduce experimental colitis in several studies we may speculate that its increased presence attenuated colitis also in our case (67). Potentially beneficial effects on colitis course could also have other metabolites that we found enriched after feeding plant protein diets. For instance, elevated phenylalanine could increase anti-inflammatory response via activation of epithelial calcium receptor (68), and similarly, increased glutamine could reduce NO and pro-inflammatory cytokines production (69). On the other hand, threonine supplementation has been shown to delay epithelial regeneration after colitis (70). Taken together, plant protein metabolites seem to have a potential to reduce colitis probably by a combined effect including strengthening of gut barrier and decreasing pro-inflammatory stimulation.

To further analyze the importance of gut microbiota in aHPD colitogenic effect, we used GF mice. Nevertheless, we did not find any significant differences in colitis severity between aCD- and aHPD-fed GF mice suggesting that the pro-inflammatory effect of aHPD is dependent on the presence of microbiota. While the absence of microbiota decreases the sensitivity of animals to acute DSS-induced colitis (71), it does not completely prevent it. This fact could even increase the sensitivity of our model to gauge the pro-colitic effect of aHPD. Next, we transferred gut microbiota from aCD or aHPD fed mice to GF mice that were not exposed to either diet and induced acute colitis in these mice. We found that mice transferred with microbiota from aHPD do not have significantly more severe colitis than these transferred with microbiota from aCD. This suggests that the both specific microbes and specific substrate (casein) needs to be present at the same time to increase sensitivity to intestinal inflammation. This is in agreement with the conclusions of the recent study of Llewellyn et al. (27). Interestingly, microbiota transferred from aHPD-fed mice made mice significantly less sensitive to colitis than microbiota from aCD. Unfortunately, we are not able to explain this counter-intuitive effect of aHPD-associated microbiota.

While they are not strictly needed for the acute DSS colitis to develop, (72), the absence or increased presence of T cells may influence its severity (71, 73). Therefore, we compared the proportion of regulatory T cells or Th17 cells in mesenteric lymph nodes of animals fed HPDs with their appropriate controls. Neither aHPD nor pHPD changed either of these T cell subsets, suggesting that T cells are not essential for the pro-inflammatory effect of aHPD. To test this hypothesis, we induced acute DSS colitis in immune-deficient RAG2^−/−^ mice on different diets. Similarly as immune-competent BALB/c mice, aHPD-fed RAG2^−/−^ mice are more sensitive to acute colitis. We found that severe colitis in aHPD-fed RAG2^−/−^ mice is associated with high levels of homeostatic cytokine IL-33 and pro-inflammatory cytokines TNF-α and IL-6. While the former is produced by damaged epithelium signaling for repair (74), possibly secondary to a higher degree of epithelial damage in aHPD-fed mice, the latter are produced in high quantities by pro-inflammatory monocytes recruited from the blood (34).

We found that colonic monocytes were significantly increased in aHPD fed mice after colitis induction. To further study the effect of this mononuclear phagocytic system on aHPD-induced colitis sensitivity, we depleted macrophages in systemic compartment with clodronate (38). We found that this intervention abrogated the pro-colitic effect of aHPD and significantly decreased the numbers of colonic Ly-6C^hi^ monocytes. Our results suggest that mainly monocytes recruited through blood stream are the drivers of aHPD-associated severe colitis whereas local macrophages have rather the anti-inflammatory regulating role. This is in line with findings that depletion of resident macrophages leads to more severe colitis (75). Interestingly, colonic neutrophils are decreased as well, and since they are not sensitive to a clodronate liposome-mediated depletion (38), this is probably caused by decrease in their recruitment due to milder colitis. Moreover, splenic monocytes in aCD fed mice were more effectively depleted by clodronate liposomes than these in aHPD fed mice probably because induction of phagocytosis in aCD fed mice, which makes them more susceptible to clodronate-induced cell depletion. In conclusion, we have shown that interactions between dietary protein of animal origin (casein) and gut microbiota increases sensitivity to intestinal inflammation. This pro-inflammatory effect is not dependent on adaptive immunity, but it is driven by activated Ly-6C^hi^ monocytes and subsequent pro-inflammatory tuning of innate immunity. Our results suggest that IBD patients may benefit from changing the source of dietary protein and outline the possibility to use specific antibiotic or probiotic treatment aimed at gut microbiota.

## Ethics Statement

This study was carried out in accordance with the recommendations of the ethics standards defined by the EU legislation on the use of experimental animals (2010/63/EU) and Czech animal welfare act. The protocol was approved by the Institute of Microbiology animal care and use committee (approval ID: 85/2015 and 108/2016).

## Author Contributions

KK and MKv designed and carried out the experiments, analyzed the data and wrote the manuscript. SC, NG, AF, TH, PP, and ZJZ helped executing the experiments and analyzed the data. MC and MKu designed, performed and analyzed the metabolomics experiments. BS carried out the statistical analysis of metabolomic data. MKo performed and interpreted the bioinformatic analysis. HT-H helped in planning experiments and discussing important concepts. All authors critically revised the manuscript for important intellectual content and approved the final manuscript.

### Conflict of Interest Statement

The authors declare that the research was conducted in the absence of any commercial or financial relationships that could be construed as a potential conflict of interest.
