# Supplementary material for: Diet Rich in Animal Protein Promotes Pro-inflammatory Macrophage Response and Exacerbates Colitis in Mice
Source: Front Immunol. 2019 Apr 26;10:919. doi: 10.3389/fimmu.2019.00919 (PMC6497971; doi:10.3389/fimmu.2019.00919)
Supplement: Supplementary file 1 [file Data_Sheet_1.pdf]

Supplementary Material

1 Supplementary Methods

1.1. NMR-based metabolomics

All NMR experiments were performed at 300 K. Standard 1H NMR spectra were acquired using Carr-Purcell-Meiboom-Gill (CPMG) pulse sequence with following acquisition parameters: number of scans (NS) = 256, 64k of data points (TD), spectral width (SW) of 20 ppm, relaxation delay (D1) of 4 s. The resonances of water were suppressed by presaturation during relaxation delay. The J-resolved experiment with presaturation (NS=4, SW=16 ppm, TD=8k, number of increments=40, SW=78.125 Hz in the indirect dimension, and relaxation delay=2 s) was performed to facilitate the identification of metabolites. All experiments were measured using standard manufacturer's software Topspin 3.2 (Bruker BioSpin, Rheinstetten, Germany).

The acquired free induction decays (FIDs) were multiplied by an exponential window function (LB = 0.3 Hz). The spectra were automatically phased, baseline corrected and referenced to TSP (0.0 ppm). The spectral baselines were adjusted according to the following two steps: to estimate the baseline within multiple shifted windows of width 200 separation units and to calculate regressions of the varying baseline to the window points using a spline approximation. Univariate statistics using Wilcoxon-Mann-Whitney test was then targeted to all individual signals, which intensities were normalized to the initial pellet weight using Matlab 3.9.14 software. The peak assignment was performed also using Chenomx NMR Suite 7.6, and the HMDB database and published assignments. The metabolite identification was supported by J-resolved and COSY experiments and confirmed using 2D NMR experiments.

2 Supplementary Figures and Tables

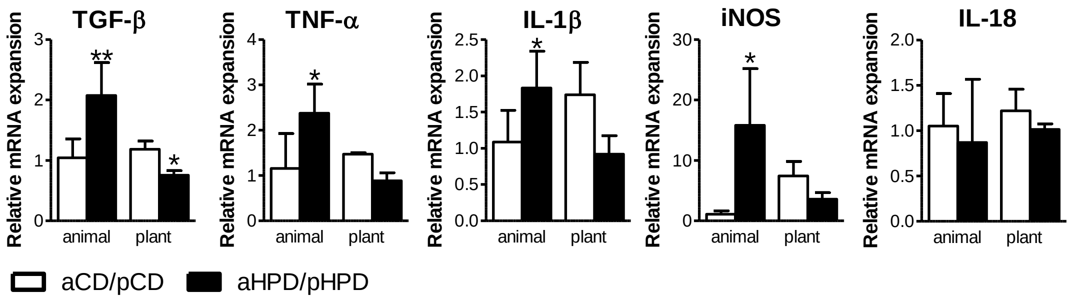

Supplementary Figure 1. In colitic mice, animal protein-based HPD increases colonic mRNA expression of TGF-β, TNF-α, IL-1β, inducible NO synthase (iNOS), but do not change the IL-18 expression. HPD of plant origin generally decreases their expression. Data are expressed as mean ± SD. All parameters were analyzed by unpaired Student's t-test; *p<0.05 **p<0.01 (n=5-8).

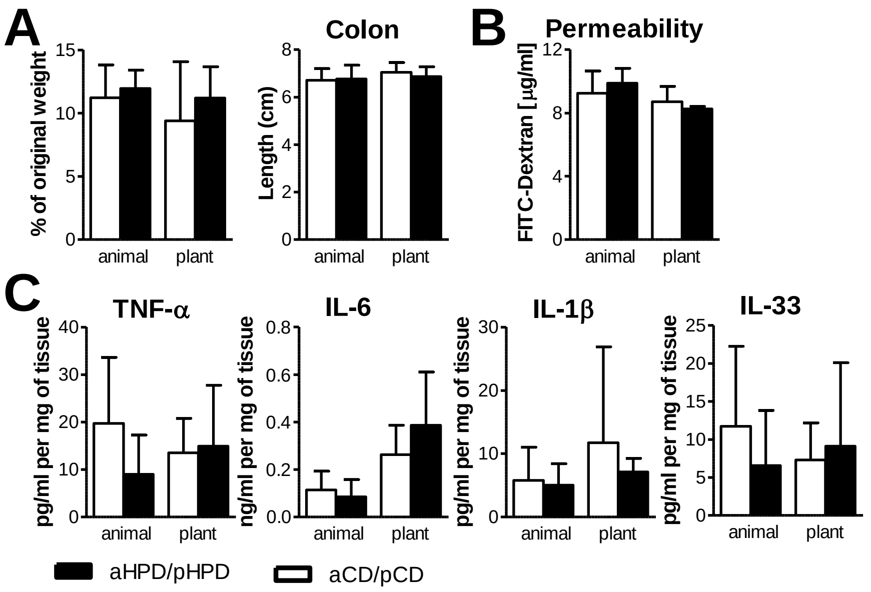

Supplementary Figure 2. Healthy BALB/c mice fed diets with different sources of protein do not have any significant differences in weight gain between day -21 and 0 or colon length (A). There were no signs of microscopic mucosa damage (data not shown). Neither HPD diet significantly changed the gut permeability for macromolecules, as analyzed *in vivo* by FITC-dextran method, (B) or production of cytokines by colon tissue, as analyzed by ELISA in tissue culture supernatants (C). Data are expressed as mean ± SD. All parameters were analyzed by unpaired Student's t-test (n=4-6).

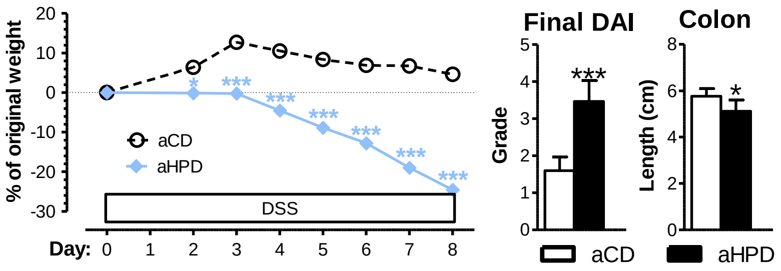

Supplementary Figure 3. If mice are exposed to it from birth, HPD of animal origin worsens the severity of acute DSS colitis. Data are expressed as mean (weight change) or mean ± SD. The weight loss was analyzed by two-way ANOVA and other parameters by unpaired Student's t-test; $*p<0.05$ $***p<0.001$ (n=5).

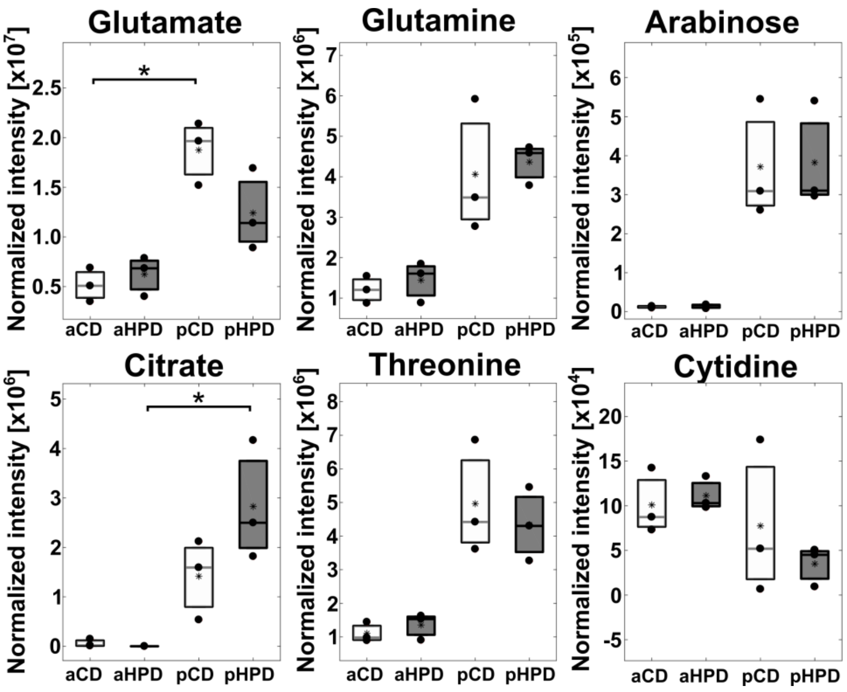

Supplementary Figure 4. Animal and plant protein diets produce different metabolites as measured by NMR-based metabolomics approach in fecal content. The biggest shifts were found in glutamate, glutamine, citrate, threonine, arabinose and cytidine. The data were analyzed using Wilcoxon-Mann-Whitney test; $*p<0.05$ (n=3). Boxplot legend: asterisk – mean value, black dots – metabolites' intensities in particular samples.

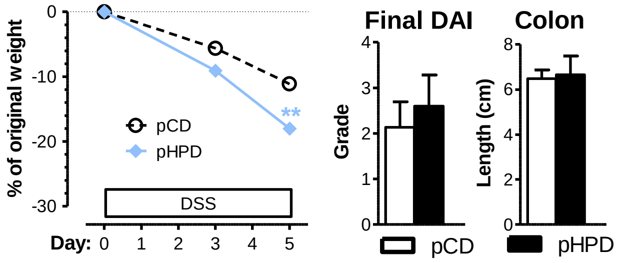

Supplementary Figure 5. In immune-deficient RAG2$^{-/-}$ mice, pHPD does not increase the sensitivity to acute DSS colitis. Data are expressed as mean (weight change) or mean ± SD. The weight loss was analyzed by two-way ANOVA and other parameters by unpaired Student's t-test; **p<0.01 (n=5).

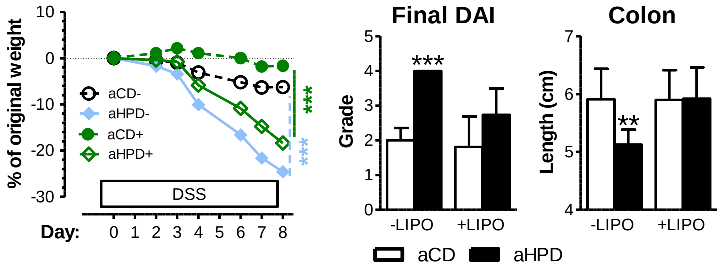

Supplementary Figure 6. Clodronate liposomes (+LIPO) prevent aHPD-induced colitis acceleration in BALB/c mice. Data are expressed as mean (weight change) or mean ± SD. The weight loss was analyzed by two-way ANOVA and other parameters by unpaired Student's t-test; **p<0.01 ***p<0.001 (n=5-8).

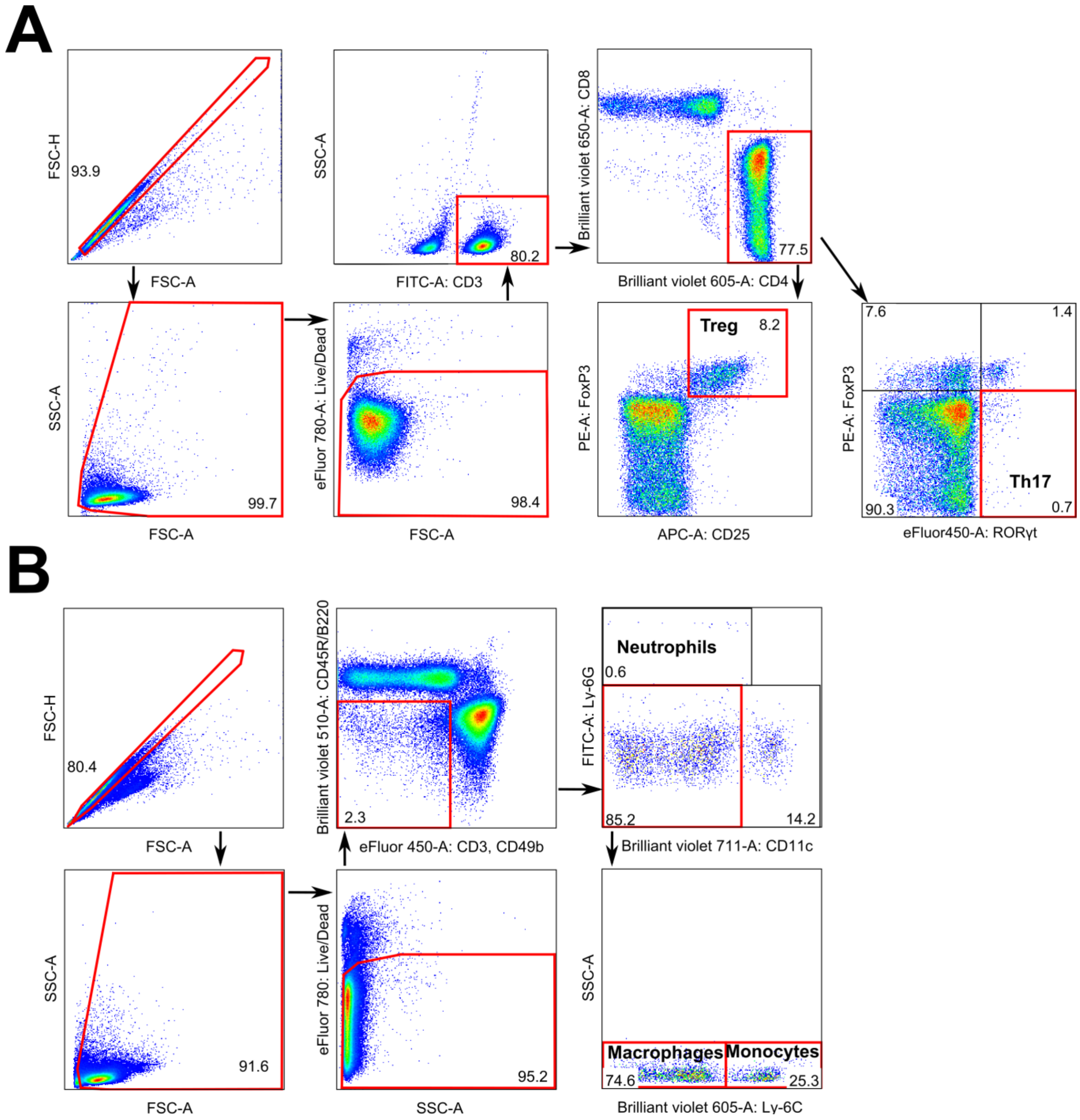

Supplementary Figure 7. Gating strategy for Treg and Th17 cells (A), monocytes, macrophages and neutrophils (B) using mesenteric lymph nodes (mLN) of colitic BALB/c mouse.

2.1 Supplementary Tables

Supplementary Table 1. Fluorochrome-labeled antibodies used for flow cytometry.

Epitope – Fluorochrome	Clone	Manufacturer	Cat#	RRID
Fixable Viability Dye eFluor 780	-	Thermo Fisher Scientific	65-0865-14	-
CD3 – FITC	145-2C11	BioLegend	100306	AB_312671
CD4 – Brilliant violet 605	GK1.5	BioLegend	100451	AB_2564591
CD8 – Brilliant violet 650	53-6.7	BioLegend	100741	AB_11124344
CD25 – APC	PC61.5	Thermo Fisher Scientific	17-0251-82	AB_469366
Foxp3 – PE	FJK-16s	Thermo Fisher Scientific	12-5773-82	AB_465936
Rorγt – Brilliant violet 421	Q31-378	BD Biosciences	562894	AB_2687545
CD3 – eFluor 450	17A2	Thermo Fisher Scientific	48-0032-82	AB_1272193
CD49b – eFluor 450	DX5	Thermo Fisher Scientific	48-5971-82	AB_10671541
CD45R/B220 – Brilliant violet 510	RA3-6B2	BioLegend	103247	AB_2561394
Ly6G – FITC	1A8	BioLegend	127606	AB_1236494
Ly6C – Brilliant violet 605	HK1.4	BioLegend	128036	AB_2562353
CD11c – Brilliant violet 711	N418	BioLegend	117349	AB_2563905

Supplementary Table 2. PCR primers used for gene expression analysis by quantitative RT-PCR.

Gene	Forward	Reverse
Rps12	CCTCGATGACATCCTTGGCCTGAG	GGAAGGCATAGCTGCTGGAGGTGT
Tgfb1	TTGCTTCAGCTCCACAGAGA	TGGTTGTAGAGGGCAAGGAC
Tnfa	TCTCTTCAAGGGACAAGGCT	CGGACTCCGCAAAGTCTAAG
Il1b	CTTCAGGCAGGCAGTATCAC	TCCATTGAGGTGGAGAGCTT
Inos	TCAGCCAAGCCCTCACCTAC	CCAATCTCTGCCTATCCGTCTC
Il18	ACGTGTTCCAGGACACAACA	ACAAACCCTCCCCACCTAAC
